# The lp13.3 genomic region -rs599839- is associated with endothelial dysfunction in patients with rheumatoid arthritis

**DOI:** 10.1186/ar3755

**Published:** 2012-03-01

**Authors:** Raquel López-Mejías, Carlos González-Juanatey, Mercedes García-Bermúdez, Santos Castañeda, José A Miranda-Filloy, Ricardo Blanco, Javier Llorca, Javier Martín, Miguel A González-Gay

**Affiliations:** 1 Department of Rheumatology, Hospital Universitario Marqués de Valdecilla, IFIMAV, Santander, Spain; 2 Cardiology Division, Hospital Xeral-Calde, Lugo, Spain; 3 Instituto de Parasitología y Biomedicina López-Neyra, IPBLN-CSIC, Granada, Spain; 4 Rheumatology Department, Hospital Universitario la Princesa, IIS-Princesa, Madrid, Spain; 5 Division of Rheumatology, Hospital Xeral-Calde, Lugo, Spain; 6 Department of Epidemiology and Computational Biology, School of Medicine, University of Cantabria, and CIBER Epidemiología y Salud Pública (CIBERESP), IFIMAV, Santander, Spain

**Keywords:** atherosclerosis, cardiovascular disease, endothelial dysfunction, rs599839, rheumatoid arthritis

## Abstract

**Introduction:**

Rheumatoid arthritis (RA) is an inflammatory disease associated with accelerated atherosclerosis and high risk of cardiovascular (CV) disease. Since genome-wide association studies demonstrated association between rs599839 polymorphism and coronary artery disease, in the present study we assessed the potential association of this polymorphism with endothelial dysfunction, an early step in atherogenesis.

**Methods:**

A total of 128 RA patients without history of CV events were genotyped for rs599839 A/G polymorphism. The presence of endothelial dysfunction was assessed by brachial ultrasonography (brachial flow-mediated endothelium-dependent (FMD)).

**Results:**

Patients carrying the allele G exhibited more severe endothelial dysfunction (FMD%: 4.61 ± 3.94%) than those carrying the wild allele A (FMD%: 6.01 ± 5.15%) (*P *= 0.08). Adjustment for gender, age at the time of study, follow-up time and classic CV risk factors disclosed a significant association between the rs599839 polymorphism and FMD (G *vs*. A: *P *= 0.0062).

**Conclusions:**

Our results confirm an association of the rs599839 polymorphism with endothelial dysfunction in RA.

## Introduction

Rheumatoid arthritis (RA) is a complex polygenic autoimmune inflammatory disease with high risk of cardiovascular (CV) complications [1]. This is a consequence of accelerated atherosclerosis [1]. Besides classic CV risk factors and chronic systemic inflammation, recent studies have emphasized the relevance of several genetic polymorphisms, such as *HLA-DRB1 *and *TNF*, in the susceptibility to CV disease in RA [2,3].

A major issue in the process of accelerated atherosclerosis in RA is the development of endothelial dysfunction, an early step in the development of atherosclerosis. An important step forward might be to identify high-risk RA patients who would benefit from active therapy to prevent clinical disease. Several noninvasive imaging techniques provide the opportunity to study the relationship of surrogate markers to the development of atherosclerosis. Among them, ultrasound techniques based on flow velocity are considered efficient ways to measure subclinical atherosclerosis. Using brachial artery ultrasonography assessment, we and others have disclosed the presence of endothelial dysfunction expressed by abnormal levels of flow-mediated endothelium-dependent vasodilatation (FMD) in patients without clinically evident CV disease who had either long-standing RA [4] or early-onset RA [5]. We also disclosed that the presence of endothelial dysfunction in RA was at least in part genetically determined [4].

Genome-wide association studies (GWAS) aimed to predict coronary artery disease (CAD) revealed several novel putative loci that may increase the risk of CAD in the general population. In this regard, the polymorphism rs599839 (A > G) seems to be associated with CAD [6,7] and with higher plasma total and low-density lipoprotein (LDL) cholesterol levels [8-10]. The non-coding rs599839 variant is located near three coding genes on chromosome lp13.3 genomic region [11]. Physically, the closest genes are *PSRC1*, encoding for "proline/serine-rich coiled coil protein 1", and *CELSR2*, encoding for the "cadherin EGF LAG seven-pass G-type receptor 2". Also, located at chromosome 1p13.3 is the *SORT1 *gene, encoding for a cell surface receptor (sortilin) with multiligand capabilities that has been implicated in insulin mediated glucose uptake. Although rs599839 is located in a non-codificant region, there is strong linkage disequilibrium between rs599839 and variants located within the 3' end of the adjacent *CELSR2 *gene, including variants with potential functional effects [7]. Although it is postulated that this protein is a receptor involved in contact-mediated communication, its specific function has not been fully clarified.

Results obtained by GWAS, need to be validated with replication studies in different cohorts to confirm these findings [12].

Taking all these considerations together we aimed to determine, for the first time, the potential role of rs599839 polymorphism in the development of endothelial dysfunction in a cohort of RA patients without clinically evident CV disease.

## Materials and methods

### Patients and study protocol

A series of 128 Spanish RA patients recruited from Lugo (NW Spain) with no previous history of CV disease were included in the present study. The study was approved by the ethics committee of the Hospital Xeral-Calde (Lugo) and a subject's written consent was obtained in all the cases. All patients fulfilled the 1987 American College of Rheumatology criteria for the classification of RA [13]. Information on the main characteristics and CV risk factors of the patients enrolled in the study is shown in Table 1.

**Table 1:** Main characteristics of the RA patients included in the study

Clinical Feature	% (n/N)
Patients	128
Main characteristics	
Age at the time of disease onset (years, mean ± SD)	50 ± 13.5
Follow-up (years, mean ± SD)	12.9 ± 7.8
Percentage of women	76.9
Rheumatoid factor positive	76.5 (98/128)
Anti-CCP antibodies positive	67.2 (86/128)
Shared epitope positive	70.3 (90/128)
Cardiovascular risk factors	
Hypertension	26.5 (34/128)
Diabetes mellitus	7.8 (10/128)
Dyslipidemia	19.5 (25/128)
Obesity	4.6 (6/128)
Smoking habit	10.9 (14/128)

Anti-CCP antibodies, anti-cyclic citrullinated peptide antibodies; RA, rheumatoid arthritis; SD, Standard deviation.

### Genotyping

DNA from patients was obtained from peripheral blood using standard methods.

The rs599839 A/G polymorphism was genotyped with TaqMan SNP genotyping assays (C____972962_10) in a 7900 HT real-time polymerase chain reaction (PCR) system, according to the conditions recommended by the manufacturer (Applied Biosystem, Foster City, CA, USA). Negative controls and duplicate samples were included to check the accuracy of genotyping.

### Brachial artery reactivity

Endothelial function was determined using high-sensitivity brachial ultrasonography according to the guidelines for the ultrasound assessment of endothelial-dependent FMD% [4,14]. B-mode scan of the right brachial artery, in a longitudinal section 2 to 12 cm proximal to the antecubital fossa, was performed in supine participants using a vascular software for two-dimensional imaging, color and spectral Doppler, an internal electrocardiogram (EKG) monitor, and a 7.5-MHz phased-array transducer Hewlett-Packard SONOS 5500 system (Hewlett-Packard, Palo Alto, CA, USA). The anterior and posterior intima-media interfaces were used to define the baseline artery diameter, calculated as the average of measurements made during four cardiac cycles at end diastole. Timing of each image frame with respect to the cardiac cycle was determined with simultaneous EKG recordings on the ultrasound system digital monitor. During image acquisition, anatomic landmarks were noted to maintain the same image of the artery throughout the study using a specific stereotactic clamp. The forearm blood pressure cuff was inflated on the ipsilateral wrist to at least 50 mm Hg above resting systolic blood pressure for five minutes, and then was released. FMD% (an increase in brachial artery diameter) was measured 30 to 60 seconds after cuff release. To assess endothelium-independent vasodilatation (NTG%), we used 400 micrograms of sublingual nitroglycerin, which acts directly on vessel smooth muscle to cause vasodilatation. NTG% was measured four minutes after nitroglycerin intake. In all cases a cardiologist (CG-J) analyzed all of the ultrasound data offline. A FMD value < 7% was considered pathologic, indicating the presence of endothelial dysfunction [14]. Intraobserver variability for FMD and NTG was 1.3% and 1.9%, respectively, based on repeat brachial ultrasonography in 32 individuals. Assessment of the endothelial function of RA patients undergoing anti-TNF-α therapy was performed 24 to 48 hours before drug administration.

### Statistical analysis

The association between the genotypes of the rs599839 polymorphism and surrogate markers of subclinical atherosclerosis was tested using unpaired t test to compare between two groups, and one-way analysis of variance (ANOVA) to compare among more than two groups. We also tested the association between these parameters and alleles using analysis of covariance (ANCOVA) adjusting for gender, age, duration of the disease at the time of the ultrasonographic study and traditional CV risk factors (hypertension, diabetes mellitus, dyslipidemia, obesity and a smoking habit).

Statistical significance was defined as *P *< 0.05. All analyses were performed with STATA statistical software 9.1 (Stata Corp., College Station, TX, USA).

## Results

Results of the comparison between the different genotypes and alleles of rs599839 polymorphism according to surrogate markers of subclinical atherosclerosis are shown in Table 2. Patients carrying the allele G exhibited more severe endothelial dysfunction (FMD%: 4.61 ± 3.94%) than those carrying the wild allele A (FMD%: 6.01 ± 5.15%). However, the difference was slightly out of the range of significance (*P *= 0.08). Likewise, values of NTG% showed a similar trend with a marginal decrease of NTG% in patients carrying the allelic variant G compared with those carrying allele A (*P *= 0.06). Also, RA patients carrying the GG and AG genotypes had lower FMD% values (1.94 ± 3.98% and 5.35 ± 3.70%, respectively) than those homozygous for the AA genotype (6.15 ± 5.42%). However, the difference did not achieve statistical significance. It was also the case when genotypes were assessed according to NTG% results (Table 2).

**Table 2:** Comparison of FMD and NTG values in RA patients according to rs599839 polymorphism

	FMD% mean ± SD (n)	*P*	NTG% mean ± SD (n)	*P*
**rs599839**				
***Allele***				
A	6.01 ± 5.15 (210)	0.08	16.95 ± 8.14 (210)	0.06
G	4.61 ± 3.94 (46)		14.52 ± 6.92 (46)	
***Genotype***				
AA	6.15 ± 5.42 (87)	0.16	17.34 ± 8.34 (87)	0.19
AG	5.35 ± 3.70 (36)		15.04 ± 7.01 (36)	
GG	1.94 ± 3.98 (5)		12.66 ± 6.99 (5)	
AG+GG	4.93 ± 3.85 (41)	0.20	14.74 ± 6.96 (41)	0.09

FMD, flow-mediated endothelium-dependent (post-ischemia) vasodilatation; NTG, flow-mediated endothelial independent (post-nitroglycerin) vasodilatation; RA, rheumatoid arthritis; SD, standard deviation

Since gender, age at the time of ultrasonography study, follow-up time and classic CV risk factors may act as potential confounders of the results derived from the ultrasonography assessment; adjustment for these potential confounders was performed. Following this procedure, a comparison of FMD and NTG values in RA patients according to rs599839 alleles in an adjusted ANCOVA model yielded a significant association between the rs599839 A/G polymorphism and FMD and NTG (G *versus *A: FMD% *P *= 0.0062 and NTG% *P *= 0.041, respectively).

## Discussion

GWA studies have become a powerful approach to rapidly identifying genetic variants that influence susceptibility to common complex diseases. Novel putative loci, such as the rs599839 A/G polymorphism (chromosome 1p13.3), that seem to increase the risk to CAD have been described by this method [6,7]. This rs599839 polymorphism has also been implicated in the presence of higher plasma total and LDL cholesterol levels [8-10].

Since GWAS have to be validated by replication studies in different cohorts and endothelial dysfunction, an early step in the atherogenesis, has been described in patients with RA, we assessed for the first time the association between the rs599839 A/G polymorphism and the presence of endothelial dysfunction in a series of RA patients without clinically evident CV disease. Interestingly, an adjusted analysis disclosed that the presence of the mutant allele G was associated with the presence of endothelial dysfunction.

Impaired FMD of the brachial artery due to endothelial dysfunction has been associated with both CV risk factors and future CV morbidity and mortality in the general population [15]. In addition, endothelial dysfunction manifested by impaired FMD was observed in both long-standing RA patients [4] and early-onset RA patients [5] without clinically evident CV disease. These observations support a potential role of FMD in establishing the presence of endothelial dysfunction as a subclinical marker of atherosclerotic disease in RA. The results derived from this study suggest a potential implication of the rs599839 polymorphism in the development of endothelial dysfunction in RA. They also support the claim of a genetic influence in the development of the atherosclerotic disease in RA.

## Conclusion

Our results showed that rs599839 polymorphism is associated with the presence of endothelial dysfunction in patients with RA.

## Abbreviations

ANCOVA: analysis of covariance; ANOVA: analysis of variance; CAD: coronary artery disease; CELSR2: cadherin EGF LAG seven-pass G-type receptor 2; CV: cardiovascular; EKG: electrocardiogram; FMD: flow-mediated endothelium-dependent (post-ischemia) vasodilatation.; GWAS: genome-wide association studies; HLA: human leukocyte antigen; LDL: Low-density lipoprotein; NTG: flow-mediated endothelial independent (post-nitroglycerin) vasodilatation; PCR: polymerase chain reaction; PSRC1: proline/serine-rich coiled coil protein 1; RA: rheumatoid arthritis; SD: standard deviation; SNP: single-nucleotide polymorphism; SORT1: Sortilin-1; TNF: tumor necrosis factor.

## Conflicting interests

The authors declare that they have no competing interests.

## Authors' contributions

RLM and MGB carried out genotyping and participated in the design of the study, data analysis and drafting of the manuscript. CGJ participated in the design of the study, acquisition of data and drafting of the manuscript. SC and RB have been involved in the interpretation of data and in revising it critically for important intellectual content. AMF participated in the acquisition and interpretation of data. JL carried out the analysis and interpretation of the data. JM made substantial contributions to conception and design of the study, acquisition of data, coordination and helped to draft the manuscript and has given final approval of the version to be published. MAG-G made substantial contributions to conception and design of the study, acquisition of data, coordination and helped to draft the manuscript and has given final approval of the version to be published. All authors have read and approved the manuscript for publication.

## Acknowledgements

We thank Rodrigo Ochoa, Sofía Vargas, M. Luisa López, M. Jesús Ibañez and Sara Olavarria for their technical assistance. This study was supported by two grants from "Fondo de Investigaciones Sanitarias" PI06-0024 and PI09/007/48 (Spain). This work was partially supported by RETICS Program, RD08/0075 (RIER) from "Instituto de Salud Carlos III" (ISCIII). MGB is a beneficiary of a grant from Fundación Española de Reumatología (FER).
